# Complement gene expression profiles are altered in the AD frontal cortex

**DOI:** 10.1002/alz70855_100157

**Published:** 2025-12-23

**Authors:** Matthew Thomas Bright, Van Dien Nguyen, Bryan Paul Morgan, Wioleta Milena Zelek

**Affiliations:** ^1^ Cardiff University School of Medicine, Dementia Research Institute, Cardiff, United Kingdom

## Abstract

**Background:**

Complement contributes significantly to pathophysiology in many neurodegenerative diseases (NDDs) including Alzheimer's Disease (AD); yet, the source of complement in the brain is poorly understood. Even multiple small changes to individual complement components can compound into significant dysregulation of the whole system. Targeting complement effectively in NDDs requires understanding complement expression in the healthy brain and how it changes in pathology.

**Method:**

We integrated single‐nucleus RNA sequencing datasets, including over 600,000 cells from 97 donor frontal cortices, 60 AD donors (36 male, 24 female) and 37 control donors (23 male, 14 female). Complement expression across nine cell types, and the impact of AD pathology, sex and age on complement gene expression was examined.

**Results:**

Microglia are the principle source of *C1Q/A/B/C* and *C3* expression in both the healthy and AD brain, with more microglia expressing higher levels observed in AD. Increases in *C1R, C1S, C5* and *C7* expression are seen in AD, predominately in fibroblasts, pericytes and astrocytes. Endothelial cells strongly expressed most complement regulators including, *CD46, CD55, CD59* and *CFH* with greater expression observed in AD. The AD risk gene *CLU* demonstrated significantly higher expression in multiple cell types however, remains predominantly expressed in astrocytes. Expression of the novel complement regulator *SRPX2* was markedly increased in AD in pericytes and fibroblasts, while slightly elevated expression of *CSMD1/2/3* is seen in neurons. No significant differences in complement expression between sexes was observed in control donors. However, in AD donors sex‐based differences were seen with female donors typically demonstrating a greater percentage of cells expressing complement. This data suggests that AD alters the normal pattern of changes in complement gene expression with age, in particular, significantly higher expression of *C1R, C1S* and *CLU* are seen in the youngest age group.

**Conclusion:**

Our comprehensive complement expression atlas for the AD and control frontal cortex reveals profound complement dysregulation in AD. There are sex specific trends in this dysregulation with females demonstrating greater dysregulation. Finally, most severe complement dysregulation in AD is observed in the youngest individuals highlighting a significant age‐dependent factor that could inform the timing and design of therapeutic interventions.

